# Pilot-Scale Controlled CO_2_ Curing System for Commercial Concrete Products and Reinforced Concrete Members

**DOI:** 10.3390/ma19163512

**Published:** 2026-08-19

**Authors:** Se-Hee Hong, Indong Jang, Hoon Moon, Gi-Joon Park, Namkon Lee, Jung-Jun Park

**Affiliations:** Department of Structural Engineering Research, Korea Institute of Civil Engineering and Building Technology, 283 Goyangdae-ro, Ilsanseo-gu, Goyang-si 10223, Gyeonggi-do, Republic of Korea; shhong@kict.re.kr (S.-H.H.); indongjang7761@kict.re.kr (I.J.); hmoon@kict.re.kr (H.M.); nklee@kict.re.kr (N.L.)

**Keywords:** CO_2_ curing, high-temperature CO_2_ curing chamber, calcium carbonate, precast concrete, reinforced concrete slab, CO_2_ uptake

## Abstract

Pilot-scale validation of controlled CO_2_ curing for reinforced concrete members remains limited. This study developed a 2400 L high-temperature CO_2_ curing chamber integrating control of temperature, relative humidity (RH), CO_2_ concentration, and pressure with real-time monitoring and automated CO_2_ regulation. Its applicability was evaluated using commercial concrete bricks and a reinforced concrete slab through mass monitoring, compressive strength testing, phenolphthalein-based carbonation assessment, thermogravimetric analysis (TGA), flexural testing, and carbonation depth measurement. Real-time mass monitoring showed a net mass gain of 50.7 g after 1 h, corresponding to 2.8% of the initial mass. The CO_2_-cured bricks achieved a compressive strength of 9.77 MPa, with a calculated CO_2_ uptake of 5.72% based on TGA. The CO_2_-cured slab exhibited a compressive strength of 41.9 MPa, comparable flexural load capacity to the steam-cured slab, and a higher ductility index of 6.86. Carbonation remained within the concrete cover without reaching the reinforcement. Within the scope of the investigated materials and curing conditions, these results demonstrate the pilot-scale feasibility of controlled CO_2_ curing for commercial concrete products and reinforced concrete members and provide a basis for further member-scale validation and process optimization.

## 1. Introduction

The Republic of Korea has committed to an ambitious 2030 Nationally Determined Contribution (NDC), aiming to reduce greenhouse gas (GHG) emissions by 40% relative to the 2018 level [1]. This policy direction has intensified the need for viable carbon mitigation strategies in emission-intensive industries, including cement and concrete production. In particular, concrete is the most widely used construction material, and ordinary Portland cement (OPC), as its primary binder, is associated with a high embodied carbon of approximately 0.8 kg CO_2_ per kg of cement [2,3]. Accordingly, reducing the carbon footprint of concrete requires strategies that address both cement-related emissions and opportunities for CO_2_ utilization during production. Two major approaches have therefore attracted considerable attention in recent years. The first approach is to reduce or eliminate the use of high-carbon binders through alternative cementitious systems, such as alkali-activated materials (AAMs) and cementless concrete [4,5,6]. These systems can partially or completely replace OPC with industrial by-products or low-carbon precursors, thereby reducing binder-related CO_2_ emissions. The second approach is to utilize CO_2_ within cementitious materials through mineral carbonation, in which gaseous CO_2_ is converted into stable carbonate phases. Therefore, CO_2_ curing offers a promising pathway for simultaneously reducing carbon emissions and integrating carbon utilization into concrete production [7]. Although concrete can passively absorb atmospheric CO_2_ through natural carbonation, this process is inherently slow and requires a substantially longer period to achieve meaningful CO_2_ uptake. Therefore, intentional mineral carbonation is necessary to accelerate CO_2_ sequestration within practical timescales and contribute to near-term emission reduction targets, such as the 2030 NDC [8,9].

The potential benefits of mineral carbonation in concrete have been increasingly demonstrated in recent studies [10]. During CO_2_ curing, gaseous CO_2_ can react with calcium-bearing phases in cementitious materials, particularly calcium hydroxide, to form calcium carbonate. This reaction not only enables CO_2_ storage in a stable mineral form, but also contributes to microstructural densification and strength development [11]. For example, Padmalal et al. [12] investigated accelerated CO_2_ curing of concrete with a water-to-cementitious material ratio of 0.45 under 10% CO_2_ concentration, 35 °C, and 70% relative humidity (RH) for 6 h, 24 h, and 72 h. It is reported that CO_2_ uptake increased with curing duration and that 72 h of CO_2_ curing substantially enhanced compressive strength compared with water curing. In addition, the effectiveness of CO_2_ curing has been shown to depend strongly on curing parameters such as temperature, RH, and CO_2_ concentration. Elevated temperature can accelerate carbonation kinetics, while moisture condition and CO_2_ availability govern the balance between reaction progression and gas transport [13,14,15]. In this regard, Jang et al. [16] optimized early-age CO_2_ curing conditions using response surface methodology and demonstrated that temperature, RH, and CO_2_ concentration interactively affect CO_2_ uptake, compressive strength, and chloride resistance. Their findings further showed that excessive CO_2_ concentration may induce a pore-blocking effect by forming a dense carbonate layer near the concrete surface, indicating that CO_2_ curing must be carefully designed rather than simply intensified. Collectively, these studies show that CO_2_ curing has evolved from a conventional curing modification into an active carbon utilization strategy for concrete. Therefore, continued research on CO_2_ curing and mineral carbonation is essential to improve both the environmental and engineering performance of concrete.

Recent studies [17,18] have demonstrated the pilot-scale application of CO_2_ curing to specific cementitious products, although its application to reinforced concrete members remains limited. In this study, a 2400 L curing chamber integrating the control of temperature, RH, CO_2_ concentration, and pressure with closed-loop CO_2_ monitoring was developed for controlled member-scale curing. It was hypothesized that controlled pilot-scale CO_2_ curing could achieve effective carbonation without compromising the required mechanical and structural performance. The effectiveness of the chamber was first examined using commercial concrete bricks through real-time mass monitoring, which allowed the time-dependent CO_2_ uptake behavior to be evaluated. The performance of the CO_2_-cured bricks was further assessed by compressive strength testing and thermogravimetric analysis to quantify strength development and CO_2_ mineralization. In addition, the chamber was applied to a reinforced concrete slab to investigate its feasibility for structural members. The flexural behavior and ductility of the slab were evaluated, and the CO_2_ penetration depth was measured to confirm carbonation at the member scale. These results provide experimental evidence for extending CO_2_ curing from material-scale applications to commercial concrete products and reinforced concrete structural members, thereby supporting the development of carbon-neutral concrete technologies.

## 2. Development of High-Temperature CO_2_ Curing Chamber

**Figure 1:**
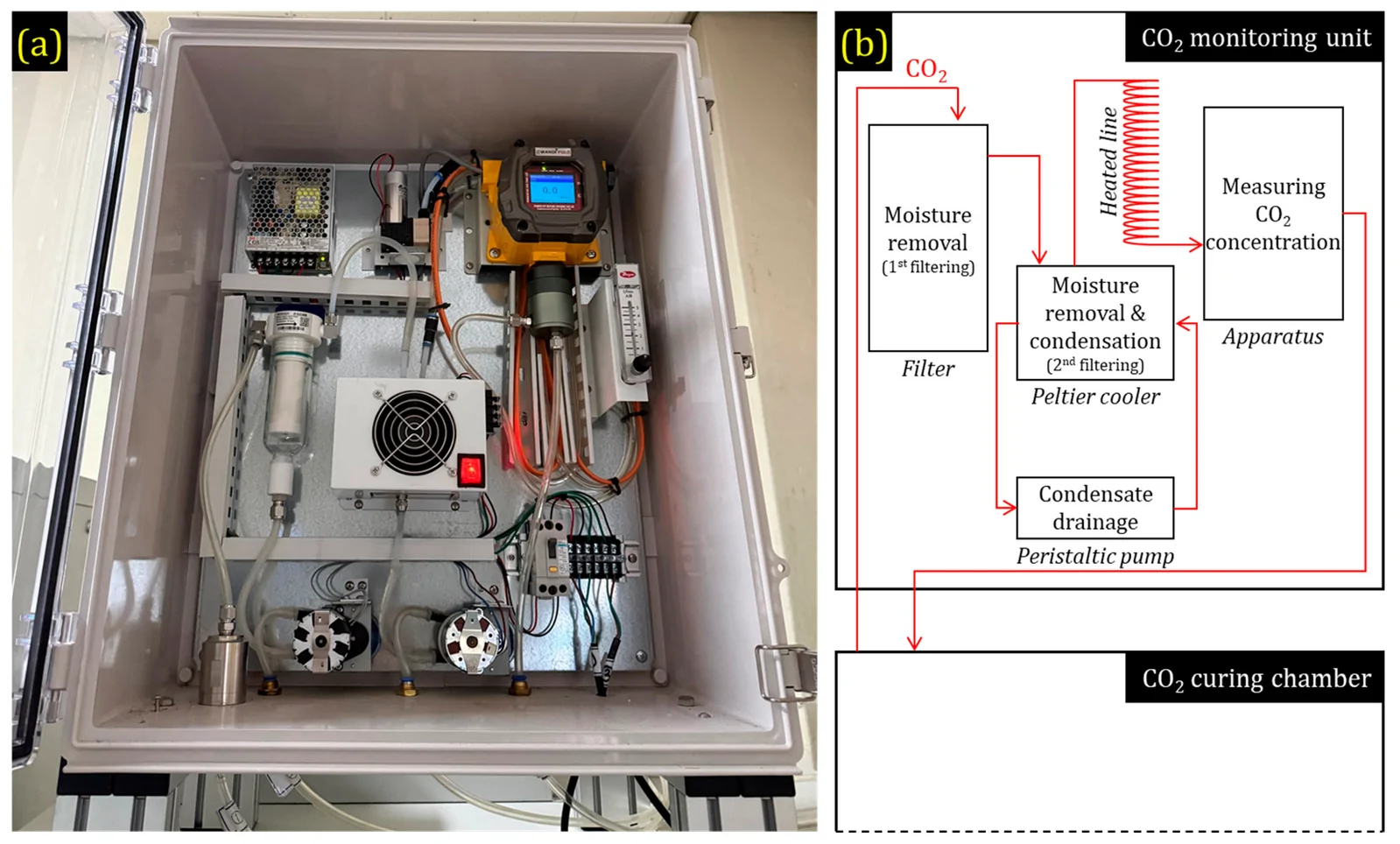
Design of closed-loop system for measuring CO_2_ concentration: (**a**) CO_2_ monitoring unit and (**b**) schematic of the closed-loop system.

The high-temperature CO_2_ curing chamber was designed to control four key operating parameters: temperature, RH, CO_2_ concentration, and pressure. To ensure stable operation and reliable monitoring, an external environmental control unit and multiple data acquisition devices were installed outside the chamber. The pilot-scale system consisted of three automated CO_2_ curing modules, each with a nominal capacity of approximately 2400 L and internal dimensions of 1.0 m × 1.0 m × 2.4 m. Each module was equipped with automated functions for CO_2_ flow measurement, CO_2_ injection, pressure relief, temperature control, humidity regulation, and real-time data logging. The CO_2_ supply was regulated using a mass flow controller (MFC), which enabled precise flow control within a range of 0–150 L/min. The CO_2_ injection system was connected to the internal CO_2_ concentration sensor, allowing an electrically actuated valve to operate automatically and maintain the target concentration. The internal pressure was also monitored continuously during curing. When the pressure exceeded the preset limit, the exhaust valve was automatically opened to prevent excessive pressure buildup and ensure safe operation. The chamber control system was configured to monitor and regulate temperature, RH, pressure, and CO_2_ concentration in real time. The measurable ranges of the control devices were 20–100 °C for temperature, 10–99% for RH, 0.01–0.5 bar for pressure, and 0–99% for CO_2_ concentration. Temperature was controlled using ceramic heaters, while humidity was supplied through a heating-wire-based water generation system. Because CO_2_ sensors are vulnerable to condensation and high-humidity exposure, a closed-loop gas sampling system was adopted for CO_2_ concentration measurement. The chamber gas was extracted through a separate sampling line and conditioned through a moisture removal system before being introduced into the CO_2_ measurement module. After measurement, the sampled gas was returned to the chamber. This configuration allowed stable CO_2_ monitoring while protecting the sensor from moisture and maintaining the internal gas balance. Figure 1 schematically illustrates the closed-loop CO_2_ sampling system used in this study.

**Figure 2:**
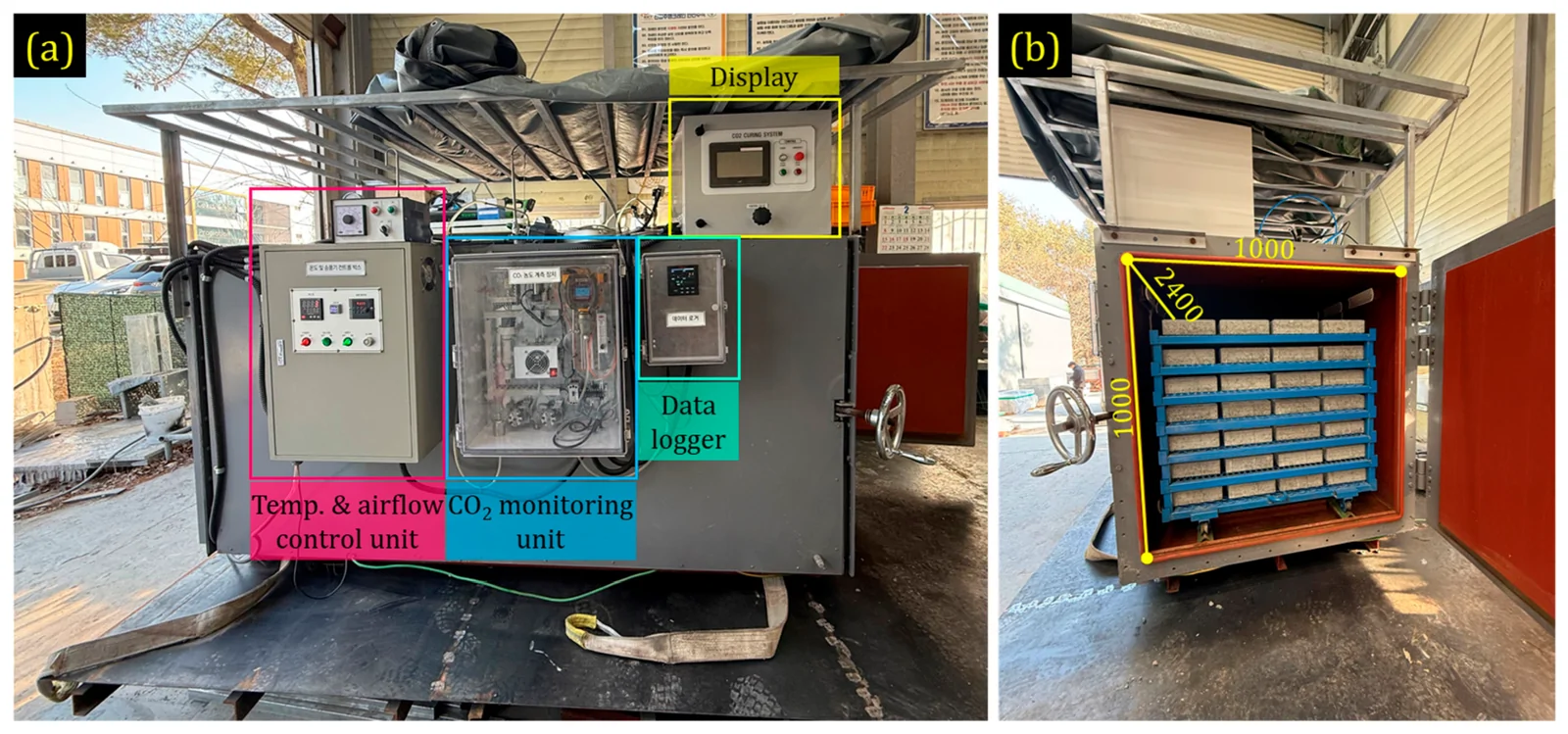
Details of high-temperature CO_2_ curing chamber: (**a**) overall system configuration and (**b**) internal chamber dimensions.

A dedicated joint design was also applied to maintain the target curing environment with high stability and to minimize CO_2_ leakage to the surrounding atmosphere. Figure 2 shows the interior and exterior views of the fabricated high-temperature CO_2_ curing chamber. A rail system was installed inside the chamber to facilitate the movement of concrete products. The chamber was designed to accommodate either a large batch of small precast concrete products or large precast elements at the member scale.

## 3. Test Program

### 3.1. Performance Assessment of the High-Temperature CO_2_ Curing Chamber

A high-precision balance was installed inside the high-temperature CO_2_ curing chamber to continuously monitor the real-time mass change in concrete bricks during curing. This measurement was used to evaluate the operational effectiveness of the chamber by tracking the mass variation associated with CO_2_ uptake and carbonation reactions. During CO_2_ curing, gaseous CO_2_ penetrates into the concrete and reacts with calcium-bearing phases to form calcium carbonate, which can result in a measurable increase in specimen mass. Therefore, the time-dependent mass change provides useful information on the overall mass variation in the brick during CO_2_ curing. However, the measured mass variation may include not only carbonation-related mass gain, but also moisture-related changes caused by evaporation, adsorption, and condensation. Because these contributions were not independently separated in the present monitoring system, the measured mass change was used as an apparent indicator of the time-dependent curing behavior, rather than as a direct quantitative measure of CO_2_ uptake. In this study, the monitoring test was conducted for 30 h under controlled conditions of 25 °C, 95% RH, and 40% CO_2_ concentration. During the test, the chamber conditions were continuously monitored and recorded to verify that the target temperature, RH, and CO_2_ concentration were maintained throughout the monitoring period. Accordingly, this approach was adopted to evaluate the real-time mass variation in commercial concrete bricks under the controlled CO_2_ curing environment.

### 3.2. Application of the High-Temperature CO_2_ Curing Chamber to Commercial Concrete Bricks

**Figure 3:**
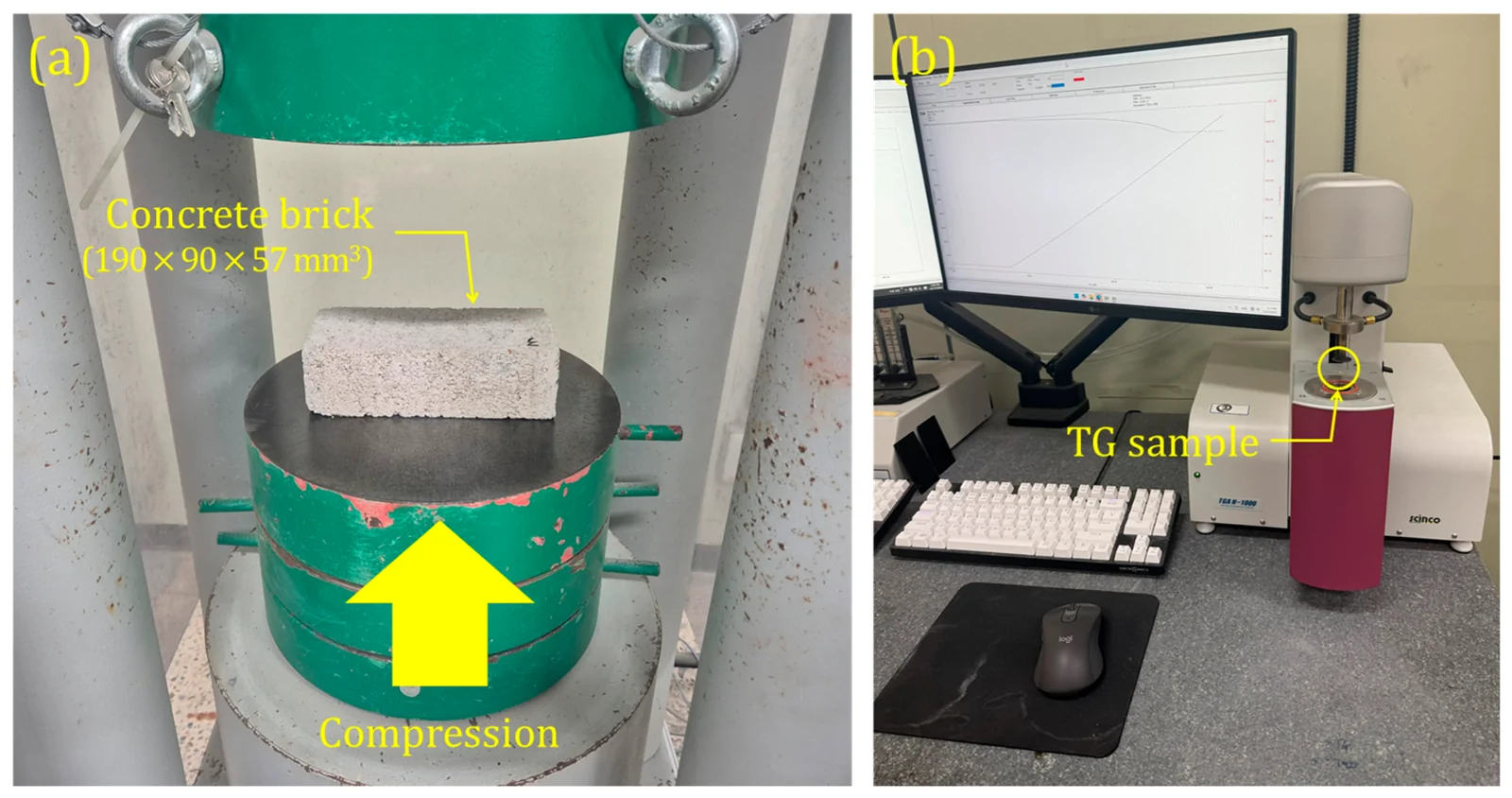
Test setups for concrete bricks: (**a**) compressive strength test and (**b**) thermogravimetric analysis (TGA).

The commercial concrete bricks used in this study had dimensions of 190 mm in length, 90 mm in thickness, and 57 mm in height, corresponding to an approximate volume of 1 L per brick. These dimensions are consistent with the commonly used standard size of concrete bricks specified in KS F 4004 [19]. To determine an efficient stacking configuration inside the high-temperature CO_2_ curing chamber, a spatial arrangement simulation was conducted. The results indicated that up to 392 bricks could be accommodated in the chamber when a clear spacing of 20 mm was maintained between adjacent bricks. For effective CO_2_ curing, the exposed surface area of the bricks should be maximized to facilitate CO_2_ diffusion and promote uniform carbonation. Therefore, the supporting tray was designed with a steel mesh structure so that CO_2_ could reach not only the side and top surfaces, but also the bottom surfaces of the bricks without obstruction. This configuration was intended to improve the uniformity of CO_2_ exposure across all faces of the bricks. In addition, the tray was designed with sufficient stiffness to safely support the total load associated with high-density stacking. Table 1 presents the mixture proportion of the commercial concrete bricks used in this study. To evaluate the effect of CO_2_ curing, two reference curing regimes, namely ambient curing and steam curing, were considered together with the CO_2_ curing condition. The steam-cured specimens were cured at 60 °C for 24 h, whereas the CO_2_-cured specimens were exposed to 40% CO_2_ at 60 °C for 72 h. The CO_2_ curing conditions were selected considering the effects of temperature, RH, and CO_2_ concentration on carbonation behavior reported in previous studies [14,16]. The curing temperature of 60 °C was also selected to provide a direct comparison with the conventional steam curing condition used for the commercial concrete bricks. After curing, the compressive strength test of the bricks was conducted in accordance with KS F 4004 [19]. Before testing, the specimens were immersed in water for 2 h to allow water absorption. The compressive strength was then determined by dividing the maximum applied load by the loaded area of the specimen. For each curing condition, three concrete bricks were tested for compressive strength, and the results are presented as the mean value with the corresponding standard deviation. After the compressive strength test, representative fractured specimens were split to expose fresh cross-sections. Phenolphthalein solution was sprayed on the exposed cross-sections to qualitatively compare the carbonation behavior of the bricks under different curing conditions. Thermogravimetric analysis (TGA) was also performed to evaluate the degree of carbonation and quantify the mineralized CO_2_ uptake. Figure 3 shows the experimental setups used for the compressive strength test and thermogravimetric analysis of the commercial concrete bricks.

### 3.3. Application of the High-Temperature CO_2_ Curing Chamber to Commercial Reinforced Concrete Slabs

**Figure 4:**
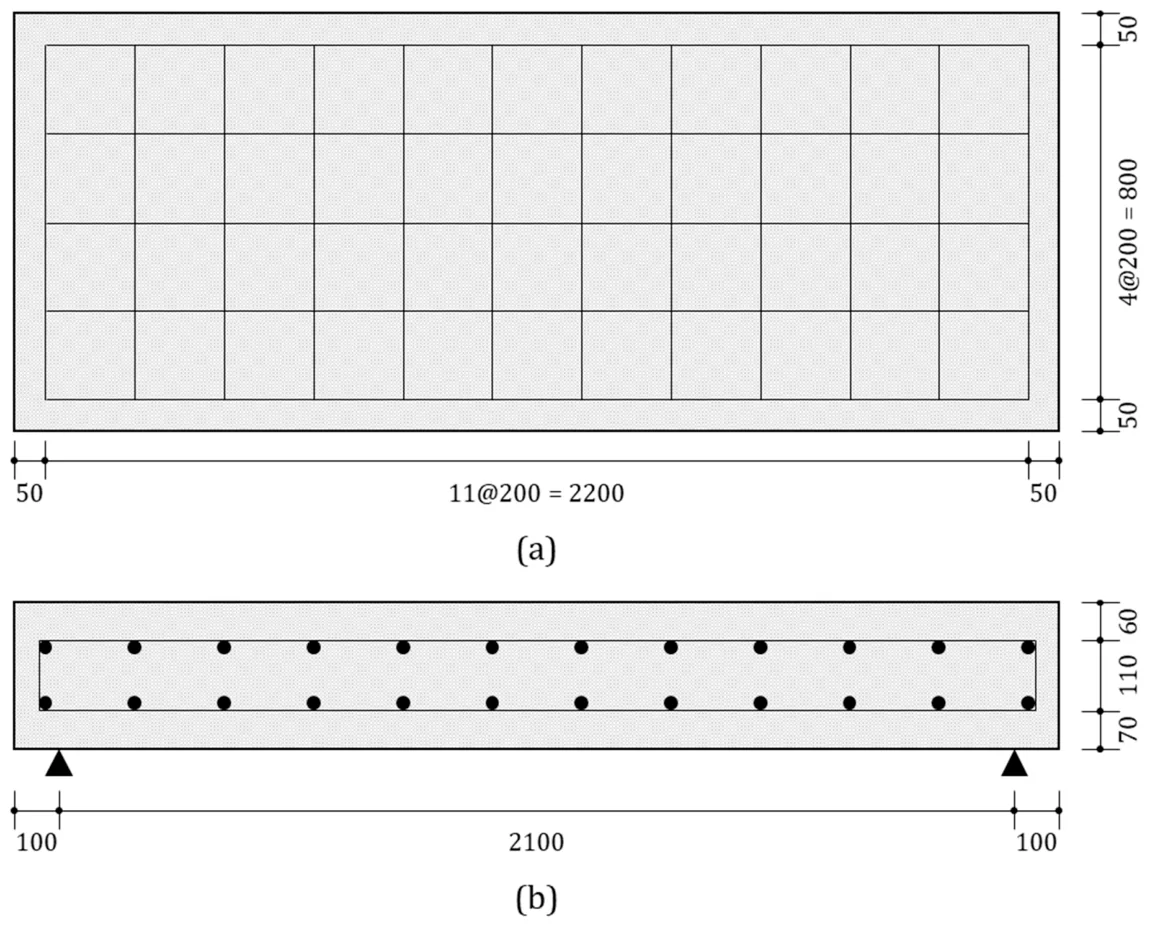
Illustration of CO_2_-cured reinforced concrete slab: (**a**) top view and (**b**) longitudinal view.

**Figure 5:**
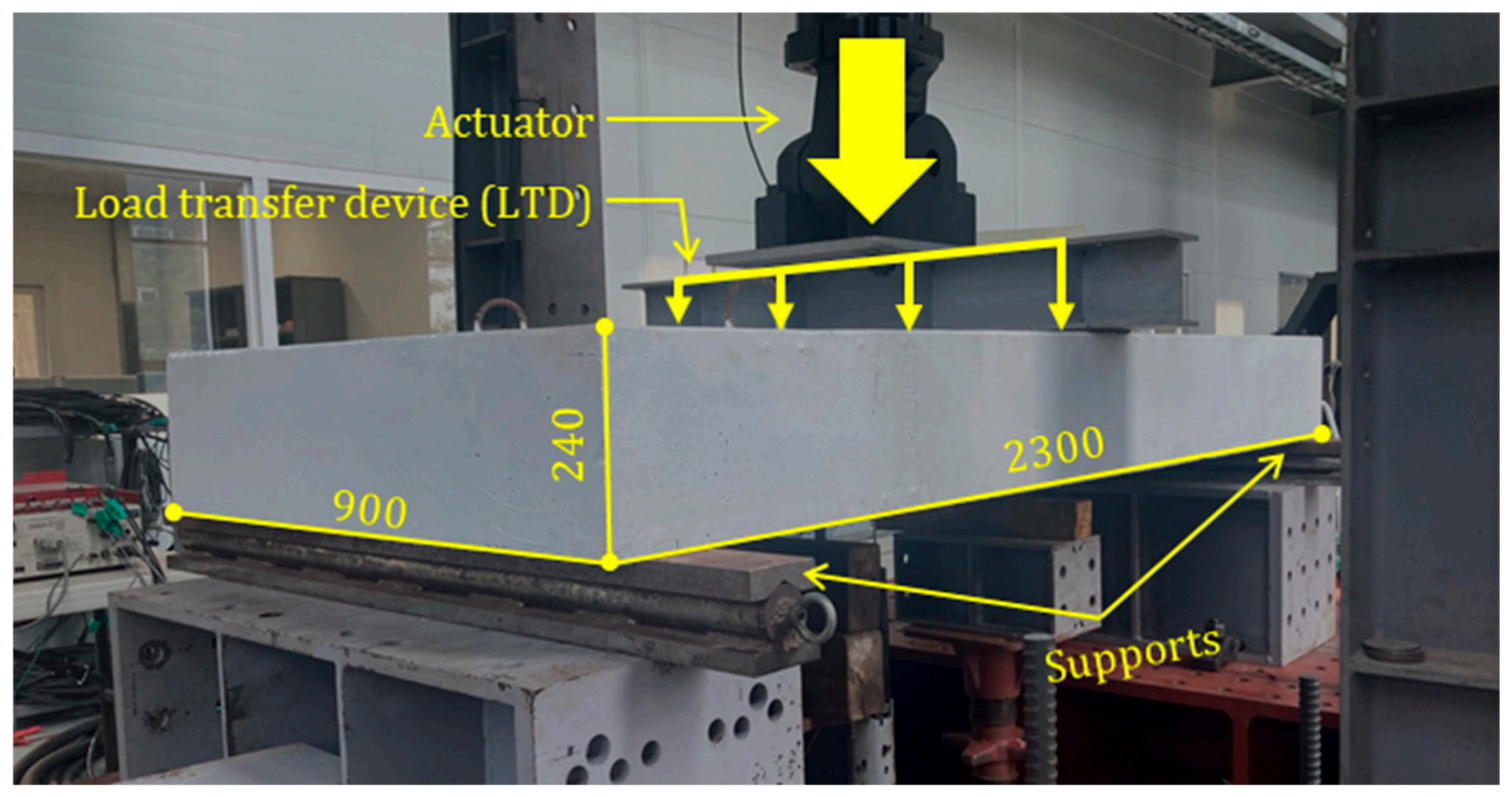
Flexural test setup of CO_2_-cured reinforced concrete slab specimen.

In this study, the applicability of the developed high-temperature CO_2_ curing chamber was extended from small commercial concrete products to a reinforced concrete slab as a representative structural member. This application was intended to examine whether the chamber could accommodate a member-scale specimen and provide a controlled curing environment suitable for evaluating the structural performance of CO_2_-cured reinforced concrete. The design compressive strength of the concrete was set to 35 MPa. Two reinforced concrete slabs were fabricated, with one slab prepared for each curing condition. A steam-cured slab fabricated with a conventional concrete mixture was prepared as a reference specimen. After demolding, the reference slab was subjected to steam curing at 60 °C for 24 h. In contrast, the CO_2_-cured slab was produced using a modified mixture in which electric arc furnace reducing slag (ERS) was incorporated as a binder component to enhance the CO_2_ storage potential of the concrete [20]. After demolding, the CO_2_-cured slab was exposed to 40% CO_2_ at 60 °C for 24 h. For each concrete mixture, the compressive strength was determined using three cylindrical specimens with a diameter of 100 mm and a height of 200 mm. The compressive strength results are reported as the mean value with the corresponding standard deviation. The mixture proportions and compressive strengths of the concretes used for the slabs are summarized in Table 2. Figure 4 illustrates the geometry and reinforcement details of the reinforced concrete slab. The slab had a width of 900 mm, a length of 2300 mm, and a thickness of 240 mm. A relatively large concrete cover was adopted from a conservative design perspective because carbonation of the cover concrete induced by CO_2_ curing may influence the corrosion resistance of reinforcing bars. All reinforcing bars used in the slab were SD400 steel with a nominal diameter of 16 mm. The bottom concrete cover was 70 mm, and the reinforcing bars were arranged at a spacing of 200 mm, as illustrated in Figure 4. The reinforcement layout, including concrete cover thickness and bar spacing, was determined based on the provisions of KDS 14 20 50 [21]. After curing, the reinforced concrete slabs were subjected to flexural testing to evaluate their load-carrying capacity and deformation behavior. Figure 5 shows the flexural test setup. A three-point loading configuration was applied using an actuator and a load transfer device (LTD), and the clear span of the slab was set to 2100 mm. A linear variable differential transformer (LVDT) with a measurement capacity of 100 mm was installed at the midspan of the slab to measure the vertical displacement during loading.

## 4. Test Results and Discussion

### 4.1. Real-Time Mass Monitoring of CO_2_-Cured Commercial Concrete Bricks

**Figure 6:**
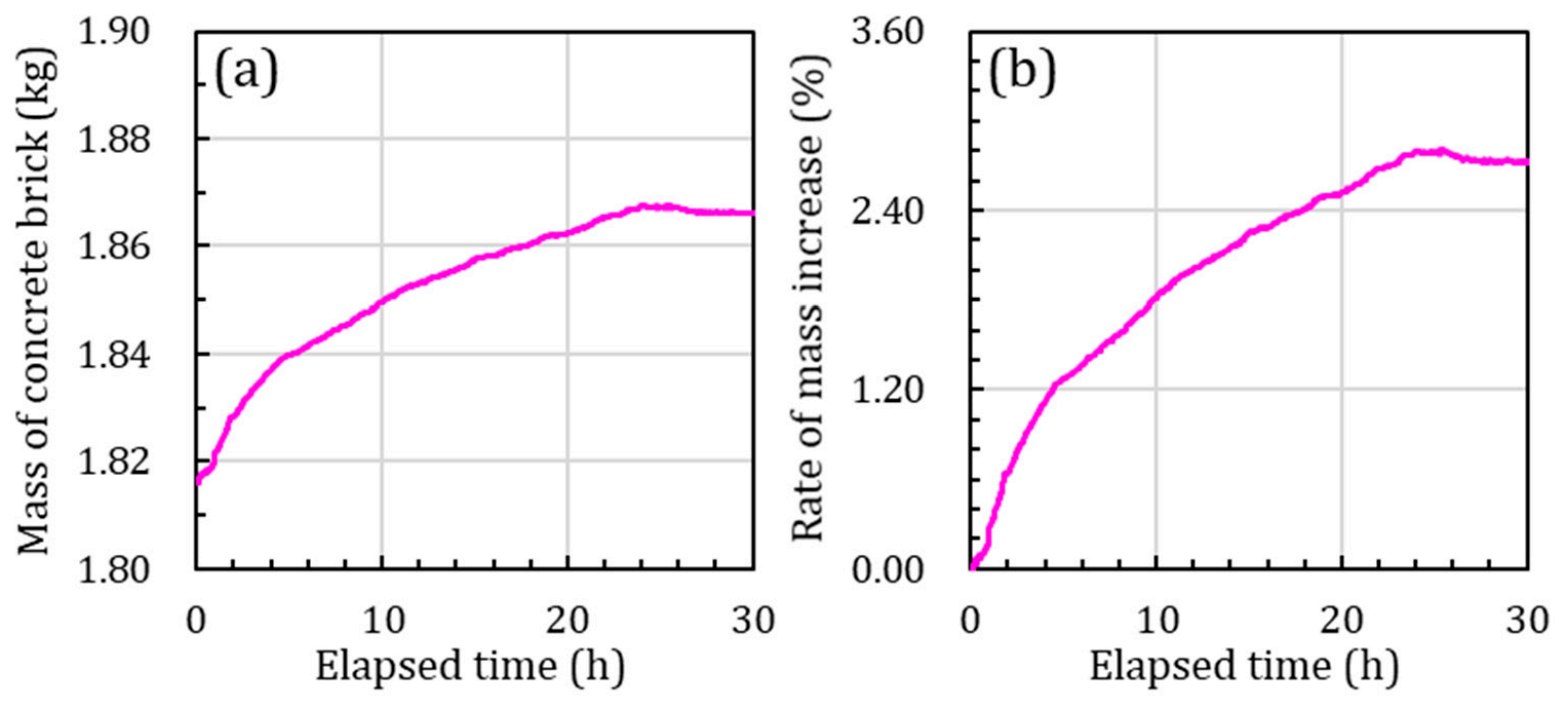
Mass variation in a concrete brick during CO_2_ curing: (**a**) specimen mass and (**b**) mass increase.

Figure 6 shows the variation in the mass of a commercial concrete brick as a function of CO_2_ curing time. The mass increased rapidly during the early stage of curing, showing a pronounced mass response under the controlled CO_2_-rich environment. After 1 h of CO_2_ curing, the specimen exhibited a net mass gain of 50.7 g, corresponding to approximately 2.8% of the initial mass. This measured mass gain may reflect carbonation reactions between CO_2_ and calcium-bearing phases in the brick, together with moisture-related changes during curing. Because the contributions of moisture adsorption, evaporation, and condensation were not independently quantified, the measured mass gain cannot be directly regarded as the amount of CO_2_ uptake. Therefore, the real-time mass monitoring result was interpreted as an apparent mass change during CO_2_ curing, while the quantitative CO_2_ uptake was independently evaluated using TGA. The mass gain exhibited a distinct stage-dependent behavior. During the initial stage, the mass increased sharply, suggesting rapid interactions among CO_2_, moisture, and reactive calcium-bearing phases near the exposed surfaces and accessible pore walls. As curing progressed, the rate of mass increase gradually decreased, which may reflect increasing diffusion limitations and the progressive consumption of readily available reactive sites near the surface. Toward the later stage of the monitoring period, the mass change became much more gradual and approached a near-plateau. This behavior may also be associated with progressive pore refinement caused by the precipitation of carbonation products. Guan et al. [22] reported that carbonation curing promoted the formation of calcite and amorphous silica, accompanied by pore refinement and microstructural densification in a belite-rich cement system. Overall, the real-time monitoring results demonstrated a distinct mass response of the commercial concrete brick during CO_2_ curing under the controlled chamber conditions.

### 4.2. Compressive Strength and CO_2_ Uptake of CO_2_-Cured Commercial Concrete Bricks

**Figure 7:**
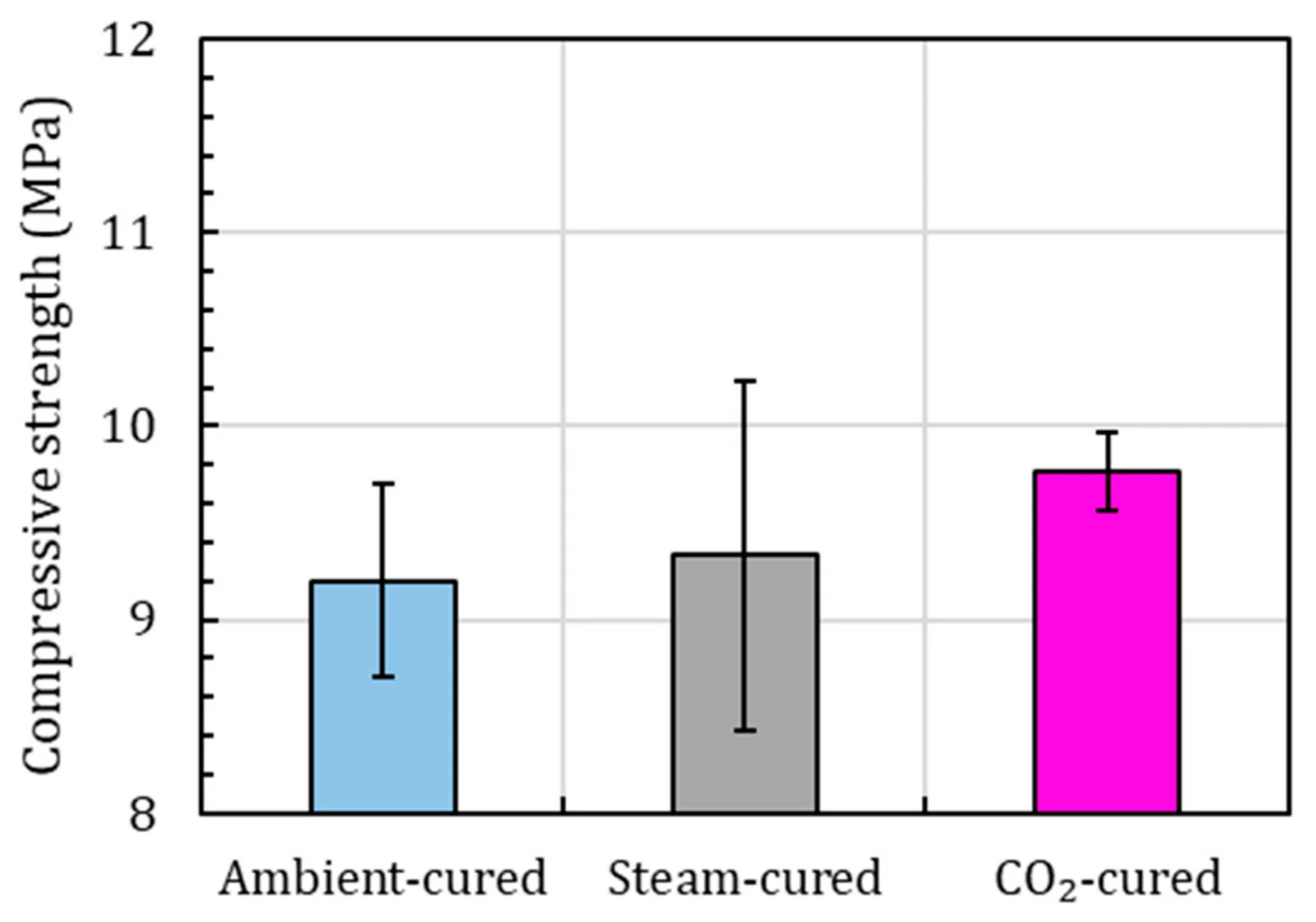
Compressive strength of commercial concrete bricks under different curing conditions.

**Figure 8:**
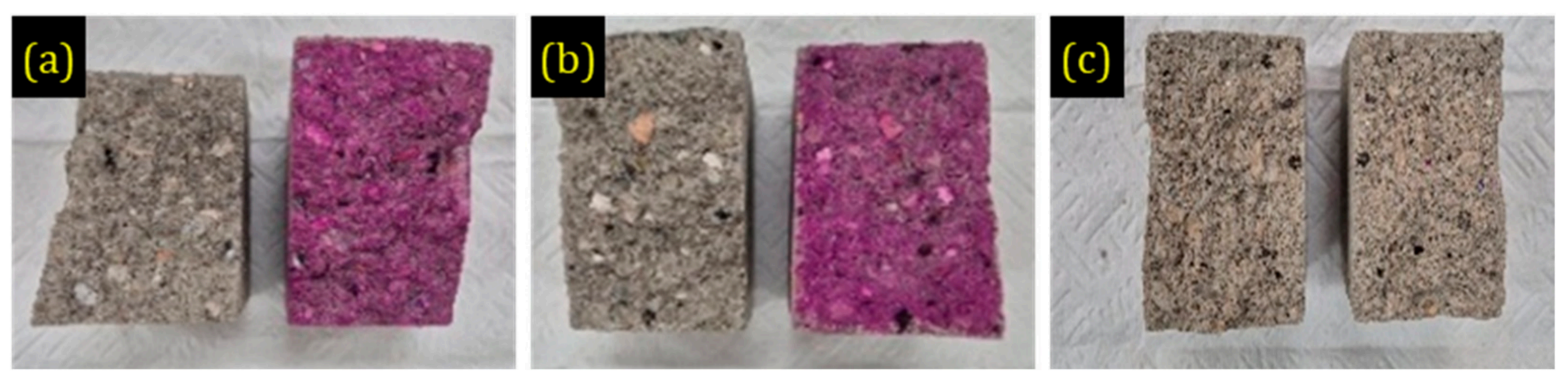
Phenolphthalein-based carbonation assessment of concrete bricks under different curing conditions: (**a**) ambient-cured, (**b**) steam-cured, and (**c**) CO_2_-cured.

**Figure 9:**
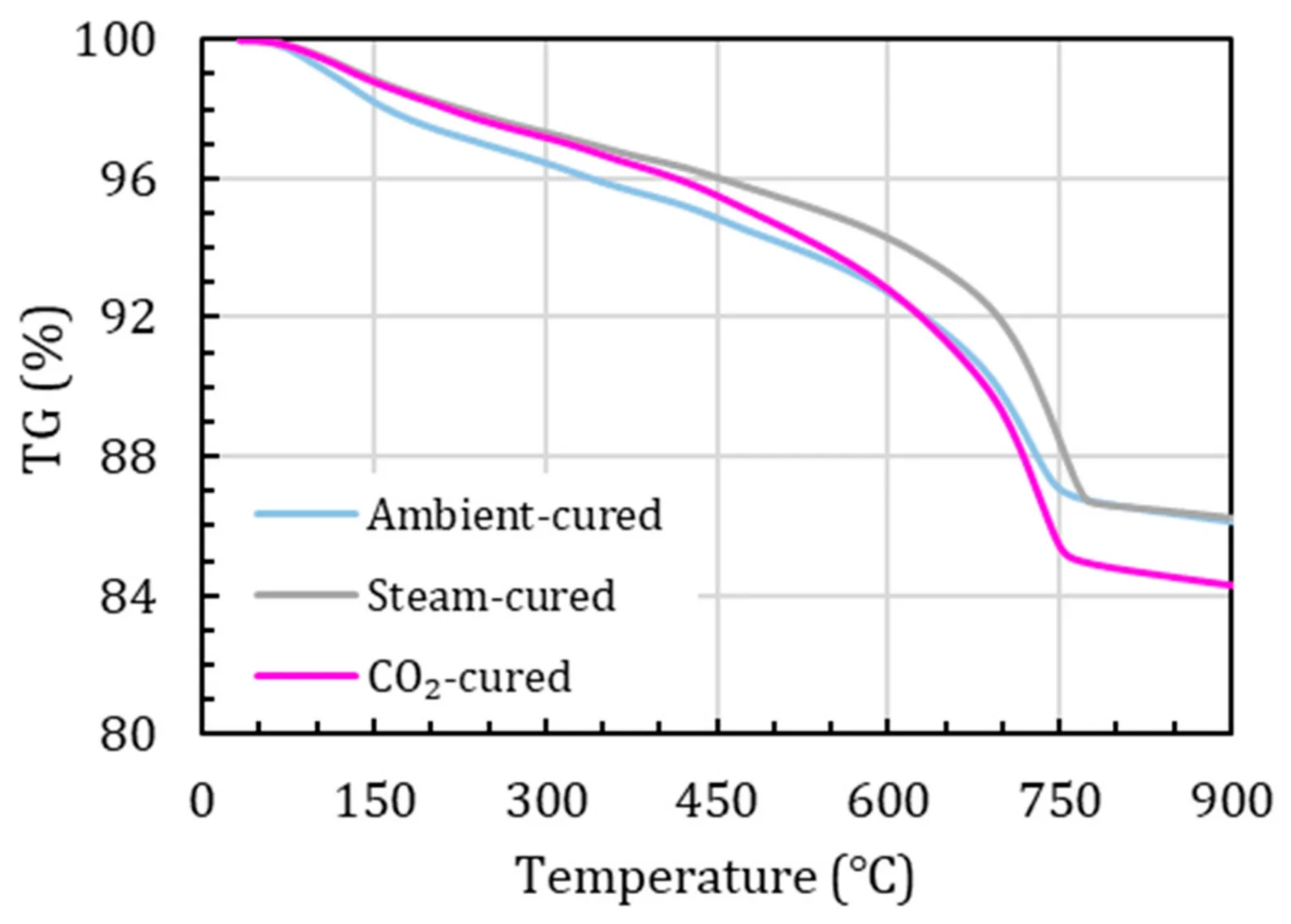
Thermogravimetric analysis (TGA) curves of commercial concrete bricks under different curing conditions.

Figure 7 compares the compressive strength of commercial concrete bricks subjected to three curing conditions: ambient curing, steam curing, and CO_2_ curing. The ambient-cured specimens showed the lowest compressive strength of 9.20 MPa. Nevertheless, all specimens satisfied the minimum compressive strength requirement of 8 MPa specified in KS F 4004 [19]. The compressive strengths of the steam-cured and CO_2_-cured specimens were 9.33 MPa and 9.77 MPa, respectively. Although the difference between steam curing and CO_2_ curing was not large, the CO_2_-cured specimens exhibited the highest average compressive strength among the investigated curing conditions. The average compressive strength of the CO_2_-cured bricks was approximately 4.7% higher than that of the steam-cured bricks. This improvement was smaller than the increase reported by Guan et al. [22] for a carbonation-cured belite-rich cement system, indicating that the magnitude of strength enhancement can vary substantially depending on the binder composition and curing conditions. This result suggests that the developed high-temperature CO_2_ curing chamber can provide mechanical performance comparable to, or slightly better than, the steam curing condition typically applied to commercial concrete bricks. In addition, the relatively small standard deviation of the CO_2_-cured specimens indicates limited variability among the three tested specimens. These results demonstrate that CO_2_ curing can be applied to commercial concrete bricks without compromising the required mechanical performance, while enabling CO_2_ utilization during the curing process. Figure 8 shows the fractured cross-sections of the commercial concrete bricks after the compressive strength test, following the application of phenolphthalein solution. Phenolphthalein is commonly used as a qualitative indicator of carbonation in cementitious materials. A strong pink color indicates a highly alkaline region, whereas a colorless region indicates reduced alkalinity caused by carbonation. In this study, the ambient-cured and steam-cured bricks showed a distinct pink color across the observed cross-sections, indicating that their internal alkalinity was largely maintained. In contrast, the CO_2_-cured brick exhibited a colorless cross-section, suggesting that carbonation progressed sufficiently to reduce the alkalinity of the brick. This behavior can be attributed to the reaction between CO_2_ and calcium-bearing phases, which consumes alkaline phases and forms carbonate products. Therefore, the phenolphthalein observation provides visual evidence that CO_2_ curing promoted carbonation within the commercial concrete brick. Figure 9 presents the TGA curves of commercial concrete bricks subjected to different curing conditions. The ambient-cured specimen showed a relatively pronounced mass loss at lower temperatures, which can be associated with the release of physically bound water and dehydration of hydration products. In contrast, the CO_2_-cured specimen exhibited a larger mass loss in the high-temperature range. In particular, the mass loss observed within the temperature range of 500–850 °C was attributed to the decomposition of carbonate phases formed through carbonation reactions [23,24]. Based on the mass loss within this temperature range, the CO_2_ uptake of the CO_2_-cured brick was calculated to be 5.72%, confirming that CO_2_ curing effectively promoted mineral carbonation under the investigated conditions.

### 4.3. Flexural Behavior of CO_2_-Cured Commercial Reinforced Concrete Slab

**Figure 10:**
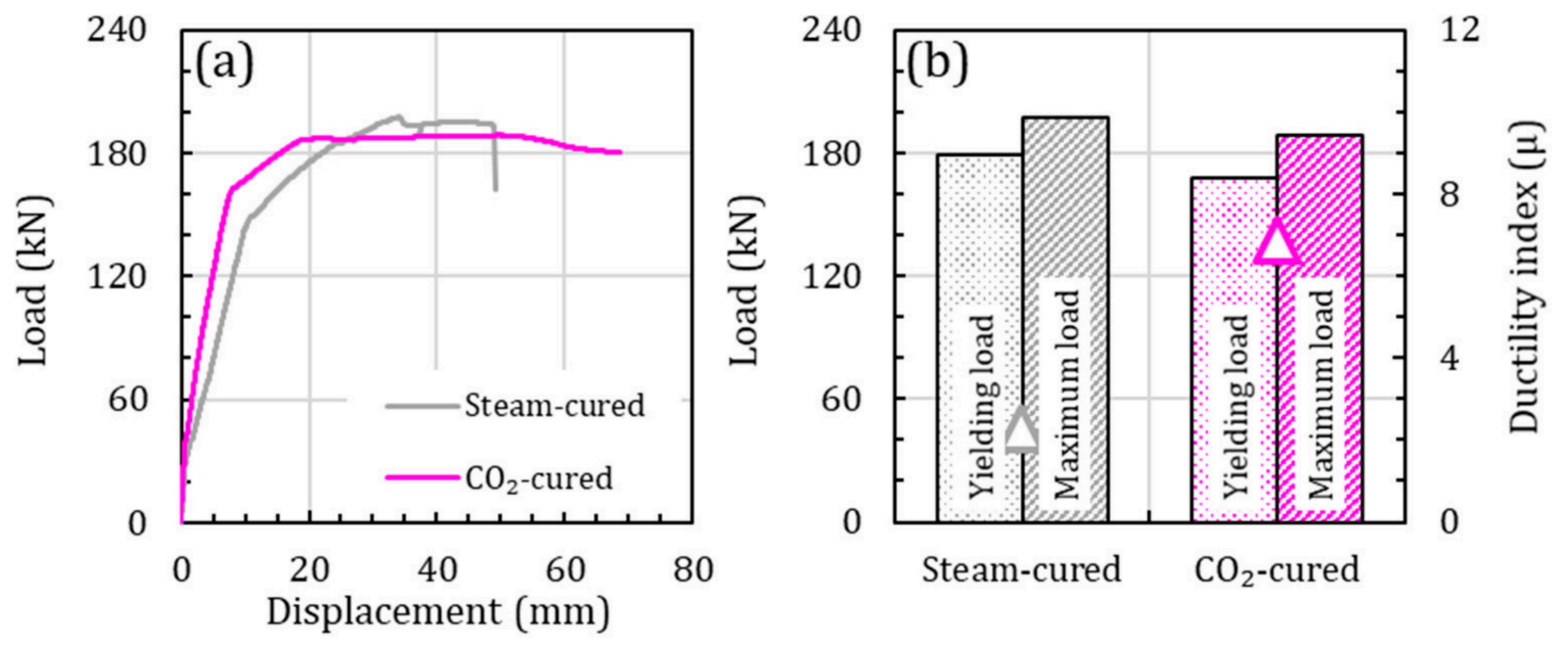
Flexural characteristics of reinforced concrete slabs under different curing conditions: (**a**) load–displacement relationship and (**b**) key flexural parameters.

The compressive strength of the concrete was measured prior to the flexural tests. The steam-cured and CO_2_-cured concretes exhibited compressive strengths of 38.0 MPa and 41.9 MPa, respectively. The higher compressive strength of the CO_2_-cured concrete suggests that the applied curing condition did not adversely affect the strength development of the concrete. However, because the CO_2_-cured concrete incorporated ERS as a binder component, the strength difference should be interpreted as the combined effect of the modified binder composition and CO_2_ curing. Accordingly, the higher compressive strength of the CO_2_-cured concrete cannot be attributed solely to the CO_2_ curing condition. Figure 10 shows the load–displacement relationships of the steam-cured and CO_2_-cured reinforced concrete slabs. In the early loading stage, both slabs exhibited an approximately linear response, indicating elastic behavior before cracking. After the formation of the first tensile crack, the load–displacement response became nonlinear due to stiffness degradation caused by crack propagation [25]. As the reinforcing bars reached the yielding range, the curves exhibited a plateau-like response. After reaching the maximum load, both slabs maintained a considerable load level while the displacement continued to increase, indicating ductile post-yield behavior. The steam-cured reinforced concrete slab showed a yield load of 179 kN and a maximum load of 197 kN. In comparison, the CO_2_-cured slab showed a yield load of 167 kN and a maximum load of 189 kN. Although the yield and maximum loads of the CO_2_-cured slab were slightly lower than those of the steam-cured slab, they corresponded to approximately 93% and 96% of the steam-cured slab, respectively. This indicates that the CO_2_-cured slab maintained a comparable load-carrying capacity under the investigated condition. The ductility index was defined as the ratio of the displacement at the ultimate state to the yield displacement, following Hong et al. [26]. This index represents the deformation capacity of a structural member and is also closely related to its ability to sustain load after yielding. As shown in Figure 10, the ductility index was 2.25 for the steam-cured reinforced concrete slab and 6.86 for the CO_2_-cured reinforced concrete slab. The ductility index of the CO_2_-cured slab was therefore approximately three times that of the steam-cured slab. This result indicates that the CO_2_-cured slab exhibited greater deformation capacity while maintaining sufficient load resistance. These findings support the potential applicability of CO_2_ curing to reinforced concrete members, particularly when both structural performance and CO_2_ utilization are considered.

**Figure 11:**
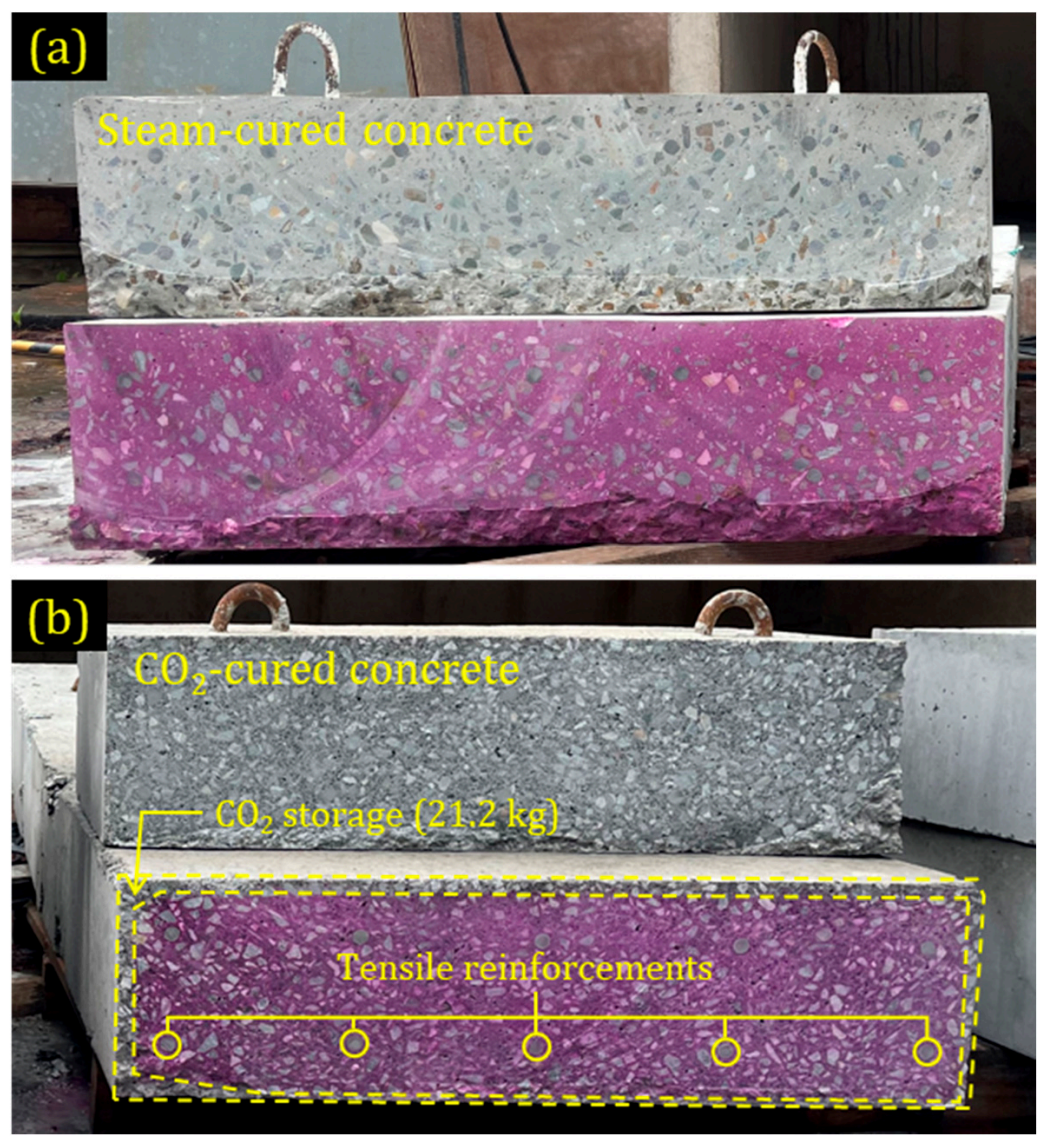
Phenolphthalein-based carbonation assessment of reinforced concrete slabs under different curing conditions: (**a**) steam-cured slab and (**b**) CO_2_-cured slab.

Figure 11 shows the cut cross-sections of the reinforced concrete slabs after the flexural tests. Phenolphthalein solution was sprayed on the cross-sections to evaluate the extent of carbonation. The steam-cured specimen exhibited a pink color across the full cross-section, indicating that carbonation was negligible. In contrast, the CO_2_-cured specimen showed a colorless region near the exposed surfaces, confirming that CO_2_ curing induced carbonation in the surface region of the structural member. The average carbonation depth of the CO_2_-cured specimen was approximately 17 mm, which was substantially smaller than the 70 mm concrete cover (Figure 4b). The carbonation front therefore remained within the cover concrete without reaching the reinforcements. The corresponding CO_2_ storage capacity was estimated to be 21.2 kg. It should be noted that the carbonation depth remained within the concrete cover and did not reach the reinforcing bars. This was achieved by providing a sufficient cover thickness in the slab design. Therefore, the CO_2_-cured slab could store CO_2_ effectively in the cover concrete while maintaining an uncarbonated region around the reinforcement. This is important because the alkaline environment around reinforcing bars is essential for preserving the passive film and reducing the risk of corrosion [27,28]. These results suggest that, with an appropriate cover design, CO_2_ curing can enable mineral carbonation within the cover concrete while maintaining comparable structural performance under the investigated conditions. Overall, the developed 2400 L high-temperature CO_2_ curing chamber was successfully applied to a reinforced concrete slab. The CO_2_-cured slab showed comparable load-carrying capacity, greater deformation capacity, and measurable CO_2_ mineralization compared with the steam-cured reference slab. These results demonstrate the feasibility of extending CO_2_ curing from small commercial concrete products to member-scale reinforced concrete elements, thereby supporting the development of carbon-neutral concrete technologies.

Compared with previous pilot-scale studies primarily focused on specific precast products [17,18], the present study extends controlled CO_2_ curing to a reinforced concrete member while maintaining comparable structural performance under the investigated conditions. From an engineering perspective, the developed chamber provides a basis for integrating controlled CO_2_ curing into precast production, although practical implementation will depend on CO_2_ supply, energy consumption for environmental control, curing duration, and production throughput. A quantitative economic assessment was beyond the scope of the present study, and therefore the economic feasibility of the proposed system remains to be established. In addition, the present findings are limited to the investigated concrete products, curing conditions, and pilot-scale structural specimens. Future studies should therefore focus on long-term durability, repeated member-scale validation, optimization of curing efficiency, and techno-economic assessment for industrial-scale implementation.

## 5. Conclusions

This study addressed the limited pilot-scale validation of controlled CO_2_ curing for reinforced concrete members by developing a 2400 L high-temperature CO_2_ curing chamber and evaluating its applicability to commercial concrete bricks and a reinforced concrete slab. The main conclusions are as follows.

(1) The developed chamber successfully provided stable CO_2_ curing conditions by controlling temperature, RH, CO_2_ concentration, and pressure. Real-time mass monitoring of the commercial concrete brick showed a net mass gain of 50.7 g after 1 h, corresponding to approximately 2.8% of the initial mass.

(2) The CO_2_-cured commercial concrete bricks achieved the highest compressive strength of 9.77 MPa, exceeding those of the ambient-cured and steam-cured specimens while satisfying the KS F 4004 [19] requirement. Phenolphthalein observation showed reduced alkalinity due to carbonation, and TGA confirmed carbonate formation with a calculated CO_2_ uptake of 5.72%.

(3) The developed chamber was successfully applied to a reinforced concrete slab at the member scale. The CO_2_-cured slab maintained comparable load-carrying capacity to the steam-cured slab, showed a higher ductility index of 6.86, and exhibited an average carbonation depth of 17 mm within the concrete cover, indicating measurable CO_2_ storage without direct carbonation of the reinforcement.

Overall, the scientific contribution of this study lies in demonstrating the feasibility of extending controlled CO_2_ curing from commercial precast products to member-scale reinforced concrete using a pilot-scale curing system with integrated environmental control. The chamber was designed to accommodate both batches of small precast products and larger precast elements, suggesting its potential applicability to a broader range of precast products, although further validation is required for different product geometries and concrete mixtures. Future studies should focus on optimizing curing conditions and process efficiency, evaluating long-term durability and reinforcement corrosion risk, validating the system across a wider range of precast products and member configurations, and conducting techno-economic assessments for industrial-scale implementation.

## Figures and Tables

**Table 1:** Mixture proportion of commercial concrete brick by cement weight.

Cement	Recycled Fine Aggregate	Stone Dust	Water to Cement (W/C)
1.0	3.3	6.7	0.5

**Table 2:** Mixture proportions of commercial reinforced concrete slab by cement weight.

Curing	Water	Cement	ERS	F.A.	C.A.	SP	Comp. Strength (MPa)
Steam	0.5	1.0	-	2.3	2.4	0.002	38.0
CO_2_	0.7	1.0	0.4	4.3	3.2	0.003	41.9

[Note] ERS: electric arc furnace reducing slag; F.A.: fine aggregate; C.A.: coarse aggregate; SP: superplasticizer.

## Data Availability

The original contributions presented in this study are included in the article. Further inquiries can be directed to the corresponding authors.

## References

[1] Lee H., McJeon H., Yu S., Liu Y., Kim H., Eom J. (2024). Decarbonization pathways for Korea’s industrial sector towards its 2050 carbon neutrality goal. J. Clean. Prod..

[2] Hong S.-H., Yuan T.-F., Choi J.-S., Yoon Y.-S. (2020). Effects of steelmaking slag and moisture on electrical properties of concrete. Materials.

[3] Meyer C. (2009). The greening of the concrete industry. Cem. Concr. Compos..

[4] Luukkonen T., Abdollahnejad Z., Yliniemi J., Kinnunen P., Illikainen M. (2018). One-part alkali-activated materials: A review. Cem. Concr. Res..

[5] Hong S.-H., Oh T., Yoo D.-Y., Park G.-J., Lee N., Park J.-J. (2026). Role of silica fume in the microstructure and mechanical properties of calcium hydroxide (CH)-activated cementless concrete. Dev. Built Environ..

[6] Amran Y.M., Alyousef R., Alabduljabbar H., El-Zeadani M. (2020). Clean production and properties of geopolymer concrete; A review. J. Clean. Prod..

[7] Saikia S.K., Rajput A.S. (2024). Effect of carbon sequestration methods on uptake potential and characteristics of ordinary portland cement-based concrete. Constr. Build. Mater..

[8] Xiao R., Prentice D., Sarkar M., Kumar A., Rosner F., Neithalath N., La Plante E., Bauchy M., Sant G. (2026). Ambient concrete carbonation is a trivial contributor in mitigating carbon dioxide emissions from cement production. Commun. Sustain..

[9] Van Roijen E., Sethares K., Kendall A., Miller S.A. (2024). The climate benefits from cement carbonation are being overestimated. Nat. Commun..

[10] Bawab J., El-Hassan H., El-Dieb A., Khatib J. (2024). Accelerated carbonation curing of concrete incorporating calcium carbide residue. J. Build. Eng..

[11] Zajac M., Irbe L., Bullerjahn F., Hilbig H., Haha M.B. (2022). Mechanisms of carbonation hydration hardening in Portland cements. Cem. Concr. Res..

[12] Padmalal A., Kulkarni K.S., Rawat P., Sugandhini H.K. (2024). Efficacy of accelerated carbonation curing and its influence on the strength development of concrete. Buildings.

[13] Drouet E., Poyet S., Le Bescop P., Torrenti J.M., Bourbon X. (2019). Carbonation of hardened cement pastes: Influence of temperature. Cem. Concr. Res..

[14] Lu B., Drissi S., Liu J., Hu X., Song B., Shi C. (2022). Effect of temperature on CO_2_ curing, compressive strength and microstructure of cement paste. Cem. Concr. Res..

[15] Xu Z., Zhang Z., Huang J., Yu K., Zhong G., Chen F., Chen X., Yang W., Wang Y. (2022). Effects of temperature, humidity and CO_2_ concentration on carbonation of cement-based materials: A review. Constr. Build. Mater..

[16] Jang I., Moon H., Park G.-J., Lee N., Park J.-J. (2026). Multi-response optimization of early-age CO_2_ curing conditions for enhanced carbon sequestration and performance of concrete. Results Eng..

[17] Zhang D., Feng B., Jaworska B., Ellis B.R., Fancy S., Sick V., Li V.C. (2025). Pilot-scale validation of CO_2_ utilization for greener and tougher infrastructure through carbonation curing of bendable concrete. J. Clean. Prod..

[18] Cho S., Lee N., Kim M.O. (2025). A comprehensive review on CO_2_ utilization in cement and concrete: From microstructural transformations to structural applications. Case Stud. Constr. Mater..

[19] (2025). Concrete Bricks.

[20] You I., Park J.-J., Lee N., Ryu G.-S., Kwark J.-W. (2024). Use of electric arc furnace oxidizing slag (EOS) and electric arc furnace reducing slag (ERS) powders in cement pastes for CO_2_ sequestrations. J. Build. Eng..

[21] (2022). Reinforcement Detail Design for Concrete Structures.

[22] Guan Q., Ma Y., Jin M., Zeng H., Gao C., Tang J., Liu J., Han F., Li W., Liu J. (2024). Carbonation curing of belite-rich cement: The role of fly ash and strengthening mechanism. Cem. Concr. Compos..

[23] Moon H., Zaheer M.H., Jang I., Park G.-J., Park J.-J., Hong S., Lee N. (2026). Utilizing carbonated reclaimed water as concrete mixing water: Improved CO_2_ uptake and compressive strength. Materials.

[24] Kim N., Choi S., Amr I.T., Fadhel B.A., Park J., Seo J., Lee H.K. (2024). Synergistic effects of CO_2_ mixing and CO_2_ curing in CO_2_ uptake capacity of Portland cement. Constr. Build. Mater..

[25] Rashid M.A., Mansur M.A. (2005). Reinforced high-strength concrete beams in flexure. ACI Struct. J..

[26] Hong S.-H., Choi J.-S., Yoo S.-J., Yoon Y.-S. (2023). Structural benefits of using carbon nanotube reinforced high-strength lightweight concrete beams. Dev. Built Environ..

[27] Xian X., Zhang D., Lin H., Shao Y. (2022). Ambient pressure carbonation curing of reinforced concrete for CO_2_ utilization and corrosion resistance. J. CO2 Util..

[28] Meng D., Unluer C., Yang E.H., Qian S. (2022). Carbon sequestration and utilization in cement-based materials and potential impacts on durability of structural concrete. Constr. Build. Mater..

